# Novel hormonal agents in men with metastatic castration resistant prostate cancer and reduced performance status: Experiences of a specialized single center

**DOI:** 10.1002/agm2.12372

**Published:** 2024-12-19

**Authors:** Thomas Büttner, Philipp Lossin, Stefan Latz, Carolin Jacobs, Philipp Krausewitz, Stefan Hauser

**Affiliations:** ^1^ Department of Urology and Paediatric Urology University Hospital Bonn Bonn Germany; ^2^ Urologie Bonn Rhein‐Sieg Bonn Germany

**Keywords:** castration‐resistant prostate cancer, ECOG, novel hormonal agents

## Abstract

**Objectives:**

Attaining castration resistance in metastatic prostate cancer (mCRPC) represents a pivotal juncture in the progression of the patient's illness and treatment regimen. Within this therapeutic context, novel hormonal agents (NHA) constitute a fundamental component of pharmacological intervention. However, the efficacy of NHA therapy remains uncertain for patients with a compromised general condition, as indicated by an Eastern Cooperative Oncology Group Performance Status (ECOG PS) score of ≥2. Notably, most clinical trials excluded individuals with an ECOG PS ≥2, leaving a gap in our understanding of the potential benefits of NHA therapy for this specific patient cohort.

**Methods:**

We conducted an analysis of fifty‐three NHA‐naïve men characterized by attaining mCRPC at an ECOG PS of ≥2 subsequent to androgen deprivation monotherapy between 2008 and 2023. Patients were then treated with either NHA or Best Supportive Care (BSC) based on individual decisions. Survival and adverse event (AE) analysis was performed to assess the outcomes of NHA therapy compared to BSC.

**Results:**

Among the patients, 30 (56.6%) received NHA, whereas the remaining 23 (43.4%) choose BSC. No significant differences in baseline characteristics were observed between the NHA and BSC group. Median overall survival (OS) was 9.1 months in the BSC group and 7.0 months in the NHA group, with no significant OS benefits associated with NHA treatment. AEs and severe AEs commonly occurred, but remained indifferent between treatment groups.

**Conclusions:**

Our findings suggest that NHA therapy may confer reduced survival benefits in mCRPC patients with ECOG PS ≥2. While hope for NHA treatment persists, particularly given its oral administration and tolerability, careful consideration and discussion with patients regarding treatment expectations and palliative care goals are warranted in this challenging patient population.

## Graphical abstract

This cohort study compared overall survival outcomes of frail men treated with novel hormonal agents for metastatic castration‐resistant prostate cancer, a special underrepresented patient cohort. Neither prolonged survival nor adverse event reduction was monitored in patients receiving treatment. We recommend considering patients' performance status in shared decision‐making and further research.

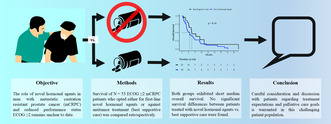

## 1 INTRODUCTION

Castration resistance in metastatic prostate cancer (mCRPC), characterized by disease progression despite adequate androgen deprivation therapy (ADT), marks a pivotal juncture in patient management.^1^ While ADT alone is initially effective and well tolerated, its efficacy wanes over time, necessitating adjunctive therapeutic approaches. Novel hormonal agents (NHA), such as enzalutamide and abiraterone, offer promising avenues by further inhibiting the androgen receptor pathway or modulating testosterone synthesis and are orally administered for convenience.^2^, ^3^, ^4^, ^5^

In mCRPC, the introduction of NHA has shown notable efficacy in tumor response, leading to prolonged overall survival (OS) and progression‐free survival (PFS). Key clinical trials such as *COU‐AA‐302* and *PREVAIL* have validated the use of abiraterone acetate + prednisolone (AA+P) and enzalutamide (ENZA) as first‐line treatment for mCRPC, highlighting both their efficacy and manageable side effects.^6^, ^7^ However, these studies primarily enrolled patients with good overall health (Eastern Cooperative Oncology Group Performance Status [ECOG PS] 0–1), leaving a knowledge gap regarding the efficacy of NHA in patients with poorer overall health (ECOG PS ≥2) which is common in the real‐world setting.^6^, ^7^

The *COU‐AA‐301* (AA+P) and *AFFIRM* (ENZA) trials enrolled patients with ECOG PS ranging from 0 to 2, but pre‐treated with docetaxel chemotherapy for mCRPC. In both trials, post‐hoc analysis of the patients with ECOG PS 2 showed that the hazard ratio for death was not significantly reduced in this subgroup compared to placebo, in contrast to the findings in men with ECOG PS 0–1.^8^, ^9^ Subsequent analyses have highlighted the importance of performance status as a predictor of patient survival across various age groups, suggesting its superiority over age alone.^10^, ^11^, ^12^, ^13^, ^14^, ^15^

Despite the growing recognition of the importance of performance status in the management of mCRPC, current guidelines from organizations such as the European Association of Urology (EAU), the European Society for Medical Oncology (ESMO), and the American Urology Association (AUA) do not provide specific recommendations regarding its role in the use of NHA for mCRPC.^1^, ^16^, ^17^ However, the EAU guideline does acknowledge that patients with ECOG PS ≥2 are less likely to benefit from drug therapy in mCRPC. In addition, the International Society of Geriatric Oncology (SIOG) defines “frail” patients based on impairment in activities of daily living (ADL), significant weight loss, or severe comorbidities, suggesting adjustment or discontinuation of mCRPC treatment in this population.^15^

Still, many patients with a compromised overall health (ECOG PS ≥2) express a desire for effective therapy, posing a therapeutic dilemma for clinicians. While NHA offers a potential solution, its efficacy in this subgroup remains uncertain. This study aims to address this knowledge gap and provide insight into the management of mCRPC in patients with ECOG PS ≥2, providing valuable information to clinicians and patients facing this complex decision‐making process.

## 2 METHODS

### 2.1 Patient cohort and ethics

Fifty‐three patients (*n* = 53) were selected from the medical records of Urology Bonn Rhein‐Sieg, an outpatient clinic where mCRPC patients are managed by independent experts (PL, SL, CJ). Comprehensive outpatient care included home visits for immobile patients requiring assistance. All patients included in this study were treated between 2008 and 2023. All had received ADT monotherapy alone for mCRPC until they progressed to castration‐resistant disease as defined by the EAU guideline. At that time, all patients had a documented ECOG PS of ≥2 according to the ECOG‐ACRIN Cancer Research Group criteria and were naïve to NHAs.^18^ They were also considered unfit for docetaxel chemotherapy. All patients signed an informed consent form authorizing the use of their clinical data with appropriate confidentiality. The study was approved by the local ethics committee (vote no. 2024‐51‐BO) and adhered to the principles of the Declaration of Helsinki.

### 2.2 Treatment groups

Choice of treatment had been made in a process of shared decision‐making and according to the current drug approval situation at the time. Patients were either treated with a first‐line oral mCRPC therapy in addition to ADT (40 mg/day ENZA or 500 + 10 mg/day AA+P, hereafter referred to as NHA group) or mCRPC‐directed treatment was not extended beyond continued ADT (hereafter referred to as BSC group). Any non‐mCRPC directed treatment with the intention to relieve symptoms or improve quality of life was allowed in both arms, e.g.; antibiotics, transfusion, osteoprotective medication.

### 2.3 Baseline data

Baseline characteristics at the initial diagnosis of prostate cancer were documented, including age, initial serum prostate‐specific antigen (PSA) levels (iPSA), metastatic sites, and details of primary prostate therapy or reasons for ineligibility for such therapy. At the time of progression to mCRPC, the following parameters were recorded and analyzed as baseline predictors of survival: age, ECOG PS, body mass index (BMI), duration of prior ADT, PSA levels, metastatic sites, serum hemoglobin (Hb) levels, Charlson Comorbidity Index (CCI), requirement of nursery care, presence of severe cognitive disorders, chronic renal insufficiency, chronic heart failure, polypharmacy, history of stroke, and use of osteoprotective medications.

### 2.4 Survival endpoints and adverse events

OS was defined as the time from the initiation of NHA therapy or best supportive care (BSC) to either death or loss to follow‐up. In addition, radiologically rPFS was evaluated in the NHA subgroup only. Differences in OS between patients treated with AA+P and those treated with ENZA and PSA response rates, defined as a ≥50% decrease from baseline mCRPC values, and time to emergency hospitalization were also analyzed. All reported adverse events (AEs) were collected, categorized based on the National Cancer Institute's Common Terminology Criteria for Adverse Events (CTCAE) v5.0, and compared between treatment groups (NHA vs. BSC, AA+P vs. ENZA).

### 2.5 Statistics

All data were coded and analyzed using RStudio 2023.09.01 + 494 (https://CRAN.R‐project.org/) package *survival*. Descriptive statistics were used, including frequencies and proportions for categorical variables, and means, medians, and ranges for continuous variables. Fisher's exact test and Student's t‐test were utilized to compare categorical and continuous variables, respectively. Survival analysis was conducted using Log‐Rank tests and the Cox proportional hazards model. Variables showing statistical significance in the univariate analysis were included in the multivariate analysis. Statistical significance was defined as *p* ≤ 0.05.

## 3 RESULTS

Of the 53 patients with mCRPC, 30 (56.6%) were treated with NHA, whereas 23 (43.4%) selected BSC. There were no significant differences in baseline characteristics between the groups except from the year of treatment, which exhibited a strong trend towards NHA treatment over time, unsurprisingly considering the approval process (Table 1). Notably, the median age at diagnosis of mCRPC was advanced with 83.8 years in the overall cohort, 83.5 years in patients receiving NHA, and 84.4 years in those treated with BSC. The majority of patients in the overall cohort had an ECOG PS 2 (71.7%), whereas the remaining patients had an ECOG PS of 3. The relative proportion of ECOG PS 2 was similar in the subgroups of NHA and BSC subgroups (73.3% and 69.6%, respectively).

**TABLE 1:** Baseline characteristics.

Characteristic	Overall *n* = 53	Best supportive care *n* = 23	New hormonal agents *n* = 30	*P*‐value^a^
Age at first diagnosis (years)				
Mean (SD)	75.2 (8.40)	75.4 (9.60)	76.8 (7.50)	0.582
Median (Range)	76.5 (51.5, 91.9)	77.1 (51.5, 91.9)	75.4 (62.4, 91.6)	
iPSA (ng/mL)				
Mean (SD)	198 (479)	221 (514)	183 (462)	0.789
Median (Range)	34.2 (1.0, 2450)	17.2 (1.0, 2180)	38.0 (4.2, 2450)	
Primary curative therapy				
Yes	14 (26.4%)	5 (21.7%)	9 (30.0%)	0.761
No – metastatic disease	26 (49.1%)	12 (52.2%)	14 (46.7%)	
No – considered unfit	12 (22.6%)	5 (21.7%)	7 (23.3%)	
Unknown	1 (1.9%)	1 (4.3%)		
Duration of ADT (months)				
Mean (SD)	65.7 (60.0)	70.7 (62.4)	62.0 59.0)	0.612
Median (Range)	43.8 (6.7, 271)	45.2 (11.1, 243)	41.8 (6.7, 271)	
Age at mCRPC (years)				
Mean (SD)	83.8 (5.9)	84.4 (6.4)	83.5 (5.6)	0.591
Median (Range)	83.9 (66.6, 96.9)	84.9 (66.6, 96.9)	83.1 (71.5, 94.1)	
PSA at mCRPC (ng/mL)				
Mean (SD)	194 (488)	176 (408)	209 (549)	0.803
Median (Range)	36.3 (0.2, 3000)	15.1 (0.2, 1500)	56.3 (3.6, 3000)	
BMI at mCRPC (kg/m^2^)				
Mean (SD)	26.4 (4.2)	25.3 (2.1)	27.1 (5.1)	0.088
Median (Range)	25.5 (18.4, 41.8)	24.8 (22.4, 30.0)	26.3 (18.4, 41.8)	
ECOG PS at mCRPC				
2	38 (71.7%)	22 (73.3%)	16 (69.6%)	0.769
3	15 (28.3%)	8 (26.7%)	7 (30.4%)	
Hemoglobin at mCRPC (g/dL)				
Mean (SD)	11.0 (1.9)	11.3 (2.1)	10.7 (1.7)	0.320
Median (Range)	11.1 (5.8, 15.4)	11.3 (5.8, 15.4)	11.1 (7.1, 13.8)	
Metastatic sites				
Bone	33 (62.3%)	13 (56.5%)	20 (66.7%)	0.511
Lymph nodes	27 (50.9%)	11 (47.8%)	16 (53.3%)	0.766
Visceral	9 (17.0%)	5 (21.7%)	4 (13.3%)	0.473
Year of treatment start				
2008–2012	17 (32.1%)	15 (65.2%)	2 (6.7%)	<0.001^**^
2013–2018	19 (35.8%)	8 (34.8%)	11 (36.7%)	
2019–2023	17 (32.1%)	0 (0%)	17 (56.7%)	
Use of osteoprotective agents				
Yes	24 (45.3%)	10 (43.5%)	14 (46.7%)	1.0
No	29 (54.7%)	13 (56.5%)	16 (53.3%)	
Charlson‐Comorbidity‐Index				
Mean (SD)	11.5 (1.2)	11.3 (1.1)	11.7 (1.2)	0.158
Median (Range)	11 (10, 15)	11 (10, 13)	11 (10, 15)	
Comorbidities				
Severe cognitive disorder	12 (22.6%)	6 (26.1%)	6 (20.0%)	0.741
Requiring nursery care	19 (35.8%)	7 (30.4%)	12 (40.0%)	0.565
Chronic renal failure	21 (39.6%)	8 (34.8%)	13 (43.3%)	0.784
Chronic cardiac failure	16 (30.2%)	7 (30.4%)	9 (30.0%)	1.0
History of Stroke	11 (20.8%)	4 (17.4%)	7 (23.3%)	0.743
Urinary catheter	24 (45.3%)	12 (52.2%)	12 (40.0%)	0.398
Polypharmacy^b^	39 (73.6%)	15 (65.2%)	24 (80.0%)	0.352

Abbreviations: ADT, androgen deprivation therapy; BMI, body‐mass‐index; ECOG PS, Eastern Cooperative Oncology Group performance status; iPSA, PSA at first diagnosis; mCRPC, metastatic castration‐resistant prostate cancer; PSA, prostate‐specific antigen in blood plasma; SD, standard deviation.

^a^ Calculated by Fisher's exact test for categorial variables and Student's *t*‐test for continuous variables.

^b^ Defined as regular intake of >5 pharmaceutical agents.

^**^ *p* < 0.001

### 3.1 Survival analysis

**FIGURE 1:**
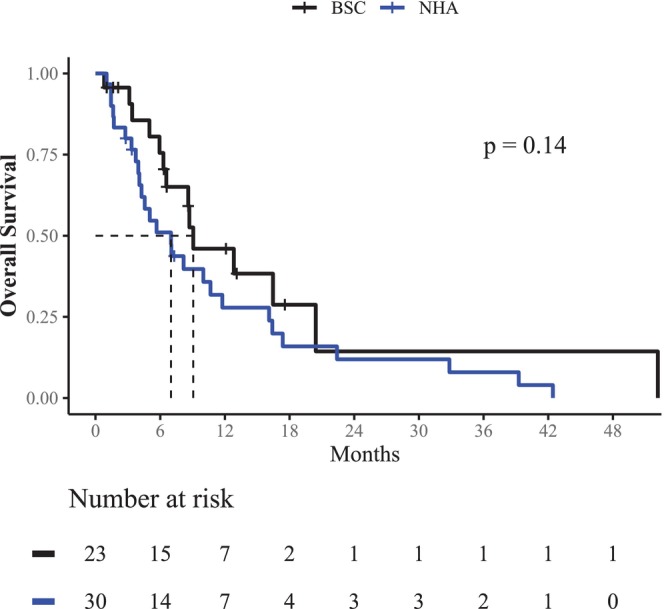
Kaplan–Meier estimators of Overall Survival (OS) in men with castration‐resistant prostate cancer and ECOG PS ≥2. Between patients treated with novel hormonal agents (NHA) or Best Supportive Care (BSC), no significant OS differences were found (median OS in NHA: 7.0 months, 95% confidence interval (95% CI): 4.3–16.1; median OS in BSC: 9.1 months, 95% CI: 6.6–NA; Log‐Rank *P* = 0.140).

The median follow‐up period was 6.6 months in the overall cohort. The estimated median OS was 9.1 months in the BSC group and 7.0 months in men treated with NHA. However, no significant OS benefits were observed for either treatment subgroup in the Cox proportional hazards model [NHA: hazard ratio (HR): 1.60; 95% confidence interval (95% CI): 0.85–3.20; *P* = 0.140] or the Log‐Rank analysis (*P* = 0.140; Figure 1). Baseline characteristics associated with OS benefits in univariate Cox regression analysis were Hb levels at mCRPC diagnosis (HR: 0.78, 95% CI: 0.64–0.95, *P* = 0.012) and metastatic disease at initial diagnosis (HR: 2.10, 95% CI: 1.10–4.10, *P* = 0.031). In multivariate Cox regression analysis, only higher Hb levels were independently predictive of prolonged OS (HR: 0.80, 95% CI: 0.66–0.97, *P* = 0.025; Table 2). Similarly, no significant differences were observed between the NHA and BSC groups in terms of time to emergency hospital admission in Cox regression analysis (HR: 1.30, 95% CI: 0.66–2.60, *P* = 0.440) or the Log‐Rank analysis (*p* = 0.420; Figure S1A). Differences in time to hospital admission, rPFS, and OS between patients treated with AA+P versus ENZA were also not significant (*P* = 0.820, *p* = 0.700, and *P* = 0.970, respectively; Figure S1B–D).

**TABLE 2:** Uni‐and multivariate cox analysis of baseline‐values and treatment choices regarding overall survival.

Univariable	Overall survival		
Characteristic	HR	95% CI	*P*‐value
Novel hormonal agent (yes vs. no)	1.6	0.85–3.2	0.140
Age mCRPC diagnosis	1.0	0.97–1.0	0.719
ECOG at mCRPC diagnosis	0.76	0.35–1.6	0.474
Duration of prior ADT	1.0	0.99–1.0	0.350
Metastatic at first diagnosis (yes vs. no)	2.1	1.1–4.1	0.031*
iPSA	1.0	1.0–1.0	0.672
PSA at mCRPC	1.0	1.0–1.0	0.466
BMI	1.0	0.94–1.0	0.950
Year of treatment: 2019–2023			*Reference*
Year of treatment: 2013–2018	0.98	0.47–2.04	0.960
Year of treatment: 2008–2012	0.81	0.36–1.79	0.602
Charlson‐Comorbidity‐Index	0.84	0.61–1.1	0.263
Severe cognitive disorder (yes vs. no)	0.85	0.39–1.9	0.687
Requiring nursery care (yes vs. no)	0.64	0.33–1.3	0.201
Chronic renal failure (yes vs. no)	1.6	0.84–2.9	0.158
Chronic cardiac failure (yes vs. no)	0.63	0.30–1.3	0.213
History of Stroke (yes vs. no)	1	0.49–2.2	0.915
Polypharmacy^a^ (yes vs. no)	0.56	0.27–1.2	0.130
Hemoglobin at mCRPC	0.78	0.64–0.95	0.012*
Osteoprotective agents (yes vs. no)	0.69	0.37–1.3	0.262
Visceral metastasis (yes vs. no)	1.8	0.75–4.3	0.188
PSA response >50% Baseline (yes vs. no)	0.67	0.20–2.3	0.517

Multivariable	Overall Survival		
Characteristic	HR	95% CI	*p*‐value
Metastatic at first diagnosis (yes vs. no)	2.0	0.96–4.2	0.06
Hemoglobin at mCRPC	0.8	0.50–0.96	0.025*

Abbreviations: 95% CI, 95% confidence interval; ADT, androgen deprivation therapy; BMI, body‐mass‐index; ECOG PS, Eastern Cooperative Oncology Group performance status; HR, hazard ratio; iPSA, PSA at first diagnosis; mCRPC, metastatic castration‐resistant prostate cancer; OS, overall survival; PSA, prostate‐specific antigen in blood plasma.

^a^ Defined as regular intake of >5 pharmaceutical agents.

* *p* < 0.05.

Of the 30 men treated with NHA, 6 patients achieved ≥50% PSA response, whereas 11 did not, and evaluable data were missing for 13 patients. However, no association with OS benefit was observed (Table 2).

### 3.2 Adverse events

AEs were common in our cohort, with over 80% of patients experiencing any grade of AE, and 73.6% experiencing severe AEs (defined as CTCAE grade ≥ 3). However, frequencies of AEs remained similar between the NHA and BSC groups (73.3% vs. 73.9%; *P* = 1.0) and between AA+P and ENZA (*P* = 1.0 for any grade AE and *P* = 0.692 for severe AE). Approximately two‐thirds of patients required hospital admission, one‐third required transfusion, and more than 10% experienced cardiac events, cognitive disorders, incidents of fall, and/or bone fractures. Nevertheless, no significant differences were identified between the NHA and BSC groups. A summary of AEs is provided in Table 3. AE‐related discontinuation of NHA was documented in 8 patients and was similar between both drugs (5 AA+P, 3 ENZA; *P* = 0.689). The median treatment duration until discontinuation was 3.4 months (range 0.5–9.2) in these patients.

**TABLE 3:** Adverse events.

Characteristic	Overall *n* = 53	Best supportive care *n* = 23	Novel hormonal agents *n* = 30	*P*‐value^a^
Any				
Grade 1–5^b^	44 (83%)	19 (82.6%)	25 (83.3%)	1.0
Grade ≥ 3	39 (73.6%)	17 (73.9%)	22 (73.3%)	1.0
Requiring Hospitalization	35 (66.0%)	15 (65.2%)	20 (66.7%)	1.0
Anemia				
Grade ≤ 2	5 (9.4%)	1 (4.3%)	4 (13.3%)	0.373
Grade ≥ 3	20 (37.7%)	11 (47.8%)	9 (30.0%)	0.252
Bone fracture				
Grade ≤ 2	4 (7.5%)	1 (4.3%)	3 (10.0%)	0.615
Grade ≥ 3	3 (5.7%)	2 (8.7%)	1 (3.3%)	0.582
Fall				
Grade ≤ 2	4 (7.5%)	3 (13.0%)	1 (3.3%)	0.309
Grade ≥ 3	7 (13.2%)	2 (8.7%)	5 (16.7%)	0.692
Infection				
Grade ≤ 2	2 (3.8%)	0	2 (6.7%)	0.503
Grade ≥ 3	14 (26.4%)	7 (30.4%)	7 (23.3%)	0.746
Cardiac events				
Grade ≤ 2	1 (1.9%)	1 (4.3%)	0	0.430
Grade ≥ 3	6 (11.3%)	3 (13.0%)	3 (10.0%)	1.0
Cognitive disorders				
Grade ≤ 2	7 (13.2%)	5 (21.7%)	2 (6.7%)	0.218
Grade ≥ 3	4 (7.5%)	3 (13.0%)	1 (3.3%)	0.307
Other				
Grade ≤ 2^c^	10 (18.9%)	4 (17.4%)	6 (20.0%)	1.0
Grade ≥ 3^d^	10 (18.9%)	3 (13.0%)	7 (23.3%)	0.484

^a^ Calculated by Fisher's exact test.

^b^ Grading by National Cancer Institute Common Terminology Criteria of Adverse Events (CTCAE) v5.0.

^c^ Gastrointestinal disorders in *n* = 4, skin disorders in *n* = 3, urinary tract dysfunctions in *n* = 2, hyperaldosteronism in *n* = 1.

^d^ Gastrointestinal disorders in *n* = 3, thromboembolic events in *n* = 2, bleeding events in *n* = 3, renal dysfunction in *n* = 2.

## 4 DISCUSSION

Our study results suggest that the survival benefit of NHA administration in mCRPC patients may be uncertain in the special group of patients with an ECOG PS of ≥2. In addition, we did not observe a reduction in AEs or emergency hospitalizations associated with NHA treatment. These results add to the limited existing evidence: Previous results from the corresponding subgroups in the COU‐AA‐301 and AFFIRM studies already suggested that an ECOG PS of 2 may influence treatment benefit, although both studies were likely underpowered for this specific subgroup analysis.^8^, ^9^ Due to the chemotherapy‐naive nature of our cohort and the inclusion of ECOG PS 3 patients, these studies also cannot be considered comparable by design. Therefore, specifically addressing the treatment of geriatric patients who have been excluded from previous study designs due to poor general health; these results are the first of their kind in a strikingly underrepresented cohort to the best of our knowledge. These results require careful evaluation and special attention as they imply that BSC may be an equal alternative to NHA for ECOG ≥2 mCRPC men.

First, the heterogeneity of mCRPC patients presents a challenge in summarizing clinical data, as demonstrated by the baseline data of our cohort. The decision for or against treatment is individualized, complex and must be tailored to the individual circumstances. Nevertheless, the increasing incidence of mCRPC in an aging society is prompting debate on this topic.^19^

It is important to recognize that many patients develop a preference for medical therapy after initial successful pretreatment for curable and/or hormone‐sensitive stages of prostate cancer prior to mCRPC. Opting for BSC in the face of progressive or even symptomatic disease can be psychologically distressing for patients, as it may be perceived as a therapeutic failure and could negatively impact their quality of life.^5^, ^20^ Given the convenience of oral administration and the availability of inexpensive generics, patients and physicians may still consider attempting NHA treatment, despite the moderate risk of drug‐related AEs.^6^, ^7^

Alternative therapeutic options to NHA are limited, particularly as patients with an ECOG PS ≥2 are frequently considered unfit for chemotherapy.^17^ Other options such as Radium‐233 or Lutetium‐177 PSMA radioligand therapy have also not demonstrated significant survival benefits in the subgroup of men with an ECOG PS ≥2.^21^, ^22^ Thus, treatment patterns similar to those described in our study are likely to persist until more effective and tolerable alternatives are developed.

While reduced treatment‐derived survival benefits in the ECOG ≥2 subgroups are thus commonly found across mCRPC and other entities, the underlying reasons have not been issue to research yet.^23^, ^24^ We suspect the reasons lie in the framework of frailty, a multisystem condition involving reduced musculoskeletal, cardiovascular, and immunological function.^25^ The ECOG PS is widely used in clinical practice and research as a standardized measure to assess the overall condition of patients, and presumably detects frailty in a straightforward manner. However, its use as a stratification tool to guide specific therapy decisions remains controversial.^26^

There are already assessment tools for mCRPC that go beyond the ECOG PS, such as the G8 screening, a SIOG‐recommended geriatric questionnaire that can predict survival and interruption of docetaxel chemotherapy in mCRPC.^15^, ^27^, ^28^ Therefore, it may also be valuable in guiding NHA treatment decisions. However, retrospective evaluation of the G8 score was not feasible in our retrospective study due to its partly subjective and patient‐reported nature. Despite this limitation, age and comorbidities, including the Charlson Comorbidity Index (CCI), showed limited prognostic utility in the current survival analysis, suggesting that an ECOG PS ≥2 alone serves as a reliable baseline assessment. It is worth noting the limitations of CCI in our cohort, particularly for patients older than 80 years with mCRPC, as they already score 10 points. Hemoglobin level remained the only prognostic factor for OS in our multivariate Cox analysis, consistent with observations made by several authors in mCRPC studies.^29^, ^30^

Further research data focusing on patients with EGOC PS ≥2 would be needed, although unlikely due to the exclusion of these patients in most prospective studies due to existing comorbidities. Still, the striking frequency of AEs and especially severe AEs in this cohort regardless of the treatment approach deserves attention. These findings demonstrate that the majority of geriatric mCRPC patients develop symptoms regardless of treatment strategy and underscore the importance of palliative support and as previously described for these patients.^31^, ^32^ Trials that focus on the needs of these patients in addition to anticancer therapy may therefore provide valuable strategies to prevent AEs and improve quality of life. And although our data are not intended or sufficient to advise clinicians to forgo NHA in patients with ECOG PS ≥2, they address the recently demanded goal of developing realistic expectations and may help to justify treatment decisions, suggesting that BSC is a valid alternative in selected individuals.^33^

### 4.1 Limitations

In addition to the retrospective design, our study has several limitations that need to be considered. These include the limited sample size at a single study center, which may be underpowered to report on significant differences. Such limitations may introduce potential selection bias, and pain scores as potential cofounders in the treatment selection process were not available for comparison. Although survival outcomes did not seem depended from the year of treatment start, treatment groups were unevenly distributed over time, encompassing potential bias. BSC lacks standardized protocols, which has been criticized by several authors.^34^ In addition, ECOG PS has been shown to have limitations in its applicability to real‐world observational data.^35^ Finally, caution is warranted when interpreting AE data, as cohort characteristics may contribute to the under‐representation of minor AEs, and due to the inherent retrospective nature precludes complete disaggregation of such data remains unattainable. Assessing how NHA treatment affects quality of life, for example through pain relief, or worsens it through the occurrence of AEs, represents a critical consideration.^31^ Unfortunately, due to the retrospective nature of our study, definitive statements regarding these effects cannot be made.

## 5 CONCLUSION

For patients presenting with a compromised ECOG PS of ≥2 in the setting of NHA‐naive mCRPC, there exists a paucity of data elucidating the survival benefit conferred by NHA therapy. In this study, no OS benefit and no reduction in AEs were observed in patients receiving AA+P or ENZA compared to BSC.

The expectations and hopes associated with NHA interventions required careful evaluation. The patient's perspective on treatment goals should be assessed and communicated by the clinician, contextualized within the individual's overall health status. The BSC framework should be discussed as an alternative, with explicit delineation and without ambiguity, to ensure optimal patient management.

## AUTHOR CONTRIBUTIONS

TB conceived the work and analyzed the datasets, prepared tables and figures, and drafted the manuscript. PL, SL and CJ acquired data. PK and SH substantially revised the manuscript draft. All authors read and approved the final manuscript.

## FUNDING INFORMATION

This study was not financially supported.

## CONFLICT OF INTEREST STATEMENT

TB received speaker honorary from Astellas and travel fees from Ipsen and MSD. PL received speaker honorary from Astellas, Apogepha, Novartis, Bayer, Ipsen, MSD, Jansen‐Cilag, BMS, travel fees from Janssen‐Cilag and Ferring and paid advisory role for Medac. PK received speaker honorary from Novartis. SH received speaker honorary from Astellas, Novartis, Bayer, Ipsen, MSD, Janssen‐Cilag, BMS, travel fees from Ipsen and paid advisory role for Ipsen, Merck and BMS. All other authors declare no conflict of interest.

## ETHICS STATEMENT

The study was approved by the responsible ethic committee of University Bonn (vote no. 2024‐51‐BO). All patients signed an informed consent form authorizing the use of their clinical data under proper confidentiality. The study was performed in accordance with the Declaration of Helsinki.

## Supporting information


Appendix S1.
