# Supplementary material for: Novel hormonal agents in men with metastatic castration resistant prostate cancer and reduced performance status: Experiences of a specialized single center
Source: Aging Med (Milton). 2024 Dec 19;7(6):761–9. doi: 10.1002/agm2.12372 (PMC11702429; doi:10.1002/agm2.12372)

**Supplementary Figure 1**: Kaplan-Meier estimators of Time to hospital admission, Progression-free Survival (PFS) and Overall Survival (PS) in men with castration-resistant prostate cancer and ECOG PS ≥2. Between patients treated with Novel Hormonal Agents (NHA) or Best Supportive Care (BSC), no significant differences were found in time to hospital admission (**A**; median in NHA: 7.3 months, 95% confidence interval (CI): 3.3 – NA; median in BSC: 8.7 months, 95% CI: 5.9 – NA; Log-Rank *P* = 0.420). Furthermore, no significant differences were found between men treated with either Enzalutamide (ENZA) or Abiraterone Acetate + Prednisolone (AA+P) in Time to hospital admission (**B**; Median in ENZA: 7.3 months, 95% CI: 3.8 – NA; median in AA+P: 3.3 months, 95% CI: 2.8 – NA; Log-Rank *P* = 0.820), Progression-free Survival (**C**; median PFS in ENZA: 4.6 months, 95% CI: 4.0 – NA; median in AA+P: 7.0 months, 95% CI: 3.1 – NA; Log-Rank *P* = 0.700) and Overall Survival (**D**; median OS in ENZA: 4.6 months, 95% CI: 4.0 – NA; median OS in AA+P: 7.0 months, 95% CI: 4.1 – 17.4; Log-Rank *P* = 0.970).


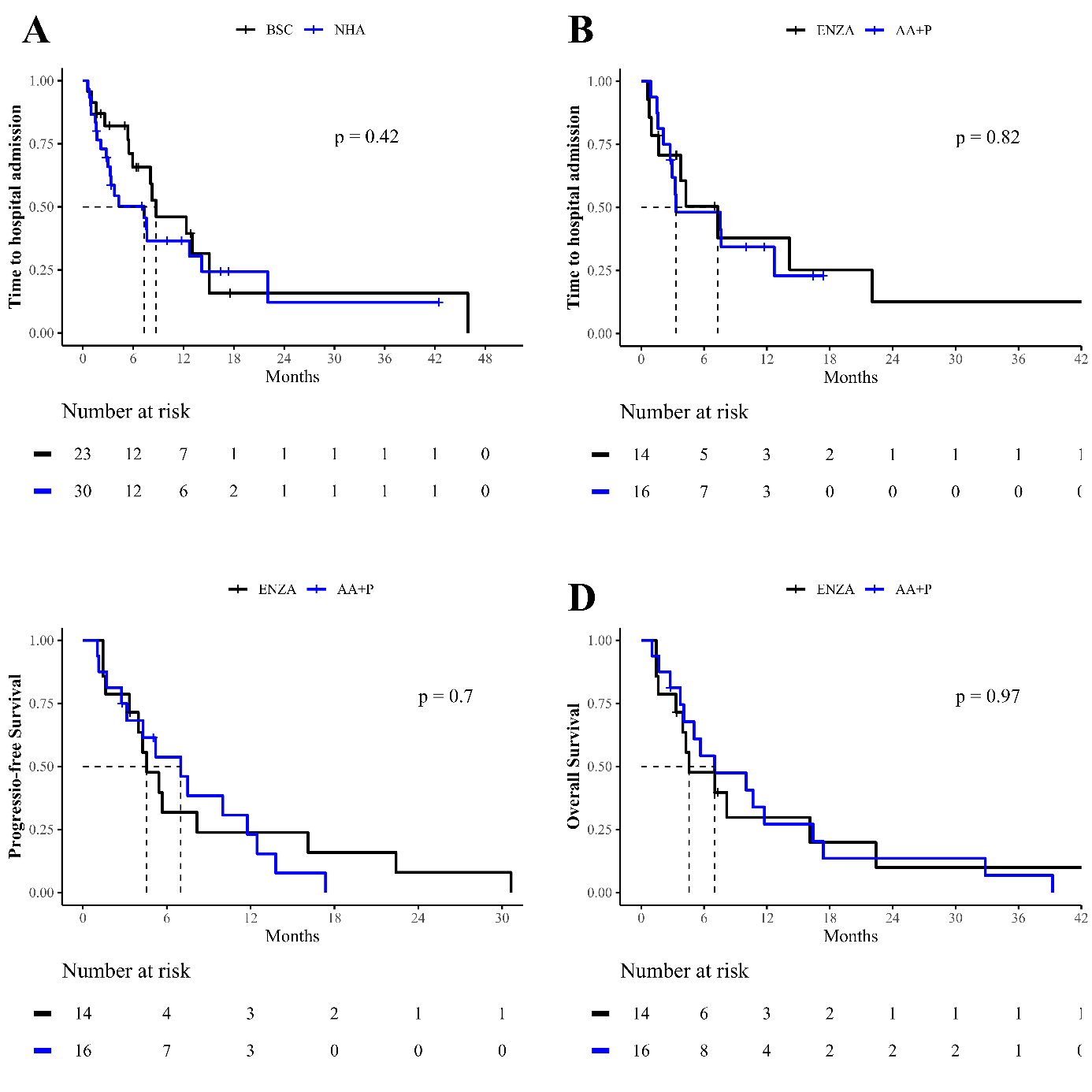

Supplement: Supplementary file 1 — Appendix S1. [file AGM2-7-761-s001.docx]
